# Changes in the gill and gut microbiota of koi infected with carp edema virus

**DOI:** 10.1186/s13567-025-01700-y

**Published:** 2026-01-08

**Authors:** Maria Zawisza, Anna Michalik, Barbara F. Nowak, Anna Pecio, Magdalena Chadzinska, Mikolaj Adamek, Krzysztof Rakus

**Affiliations:** 1 Department of Evolutionary Immunology, Institute of Zoology and Biomedical Sciences, Faculty of Biology, Jagiellonian University, Krakow, Poland; 2 Doctoral School of Exact and Natural Sciences, Jagiellonian University, Krakow, Poland; 3 Department of Invertebrate Development and Morphology, Institute of Zoology and Biomedical Research, Faculty of Biology, Jagiellonian University, Krakow, Poland; 4 Institute for Marine and Antarctic Studies, University of Tasmania, Hobart, TAS Australia; 5 Department of Comparative Anatomy, Institute of Zoology and Biomedical Research, Faculty of Biology, Jagiellonian University, Krakow, Poland; 6 Fish Disease Research Unit, Institute for Parasitology, University of Veterinary Medicine Hannover, Hannover, Germany

**Keywords:** Carp edema virus, CEV, koi sleepy disease, KSD, common carp, microbiota, gill disease

## Abstract

Carp edema virus (CEV) is a contagious pathogen affecting mostly the gills of common carp (*Cyprinus carpio*) and impairing the functions of this organ. Moreover, CEV was reported to cause stress activation and modulation of the hosts immune system. Last but not least, secondary bacterial infections often occur during CEV infection. In this study, we assessed the impact of CEV infection on the microbiota in the gills, foregut, and hindgut of koi. Moreover, we studied expression of genes encoding antiviral proteins, proinflammatory cytokines, mucins, and tight junction proteins. We observed increased levels of cortisol and glucose in the blood plasma of CEV-infected fish, suggesting activation of stress response. Histopathological analysis of the gills showed occlusion of the intralamellar space with the presence of apoptotic cells in CEV-infected fish, while in the gut only minor histopathological changes were recorded. Microbiota of the gills was significantly altered during infection with increased absolute abundance of several bacteria genera, including opportunistic bacteria e.g. *Chryseobacterium, Flavobacteria, Aeromonas*, and *Pseudomonas*. In the foregut, we did not observe changes in the absolute bacterial abundance between control and CEV-infected fish, while in the hindgut only absolute abundance of *Aeoromonas* was increased in infected fish. Moreover, CEV infection differentially altered expression of studied genes, including for example upregulation of expression of *mxa, vig, il-1b*, and *cldn 7* in studied organs. Our study explores the effect of CEV infection on the host microbiota, which may partly explain the occurrence of secondary bacterial infections during infection with this virus.

**Supplementary Information:**

The online version contains supplementary material available at 10.1186/s13567-025-01700-y.

## Introduction

Koi sleepy disease (KSD) is a serious disease affecting common carp (*Cyprinus carpio*), including its ornamental variety—koi. KSD has been known since 1970s, when it caused mass mortality among Japanese koi populations [1]. The etiological agent of the disease is carp edema virus (CEV), a poxvirus targeting primarily gill epithelium [1, 2]. Observed histopathological changes in the gills include hypertrophy and hyperplasia of epithelial cells, fusion of gill lamellae, and occlusion of interlamellar spaces [2, 3]. Damaged gills leads to impairment of the functions performed by this organ, e.g. disturbance of respiratory function, acid–base regulation, osmoregulation, and nitrogenous waste excretion [3]. This results in the hypoxia, hyponatremia, and hyperammonemia in CEV-infected fish [3, 4]. Our previous studies showed that CEV infection and its outcomes can induce a stress response. We demonstrated upregulation of the expression of stress response genes in the nucleus preopticus (NPO) of hypothalamus and an increased concentration of cortisol and glucose in the blood plasma [4]. Moreover, immunomodulatory effect of CEV infection was observed, in particular downregulated expression of genes encoding T-cell markers (*tcra1, tcra2, cd4, cd8a1, cd8b1, cd8b2, cd3)* and *zap70* [4]. In addition, CEV infection is often associated with the secondary bacterial infections which are observed during KSD. Since stress or infection-induced changes in the fish might make them more susceptible towards secondary bacterial infections, we decided to study changes of microbial community composition during KSD in koi.

Stress and infection can significantly impact the microbial community composition in fish, leading to changes in the balance of beneficial and harmful bacteria [5–10]. Increased stress levels, caused by different factors e.g. infections, poor water quality, or overcrowding, can weaken the immune response and create an environment where opportunistic pathogens thrive. For example, it was observed that chronic stress affected the relative abundance of intestinal microbiota in gibel carp (*Carassius gibel*), and α-diversity in the stressed fish was significantly lower than that in the control [11]. In Atlantic salmon fry (*Salmo salar*) stress was associated with decreased abundance of beneficial lactic acid bacteria *Carnobacterium* sp. and increased abundance of bacteria from Clostridia and Gammaproteobacteria classes [12].

Another important factor disrupting fish microbiota are infections, including viral ones. It was observed that spring viremia of carp virus (SVCV) infection disrupts microbiota of common carp in the nose and pharynx [13], as well as in the buccal mucosa, skin, and gills [14]. SVCV infection in zebrafish (*Danio rerio*) decreased bacterial diversity in the gut, alongside higher relative abundance of opportunistic bacteria [15]. Infection-related microbiota changes resulted in higher abundance of opportunistic bacteria were also observed in rainbow trout (*Oncorhynchus mykiss*) infected with infectious hematopoietic necrosis virus (IHNV) [16] and in orange-spotted grouper (*Epinephelus coioides*) infected with nervous necrosis virus (NNV) or grouper iridovirus (GIV) [17]. Recent analysis of microbiota of CEV-infected koi showed an increase of *Flavobacterium* [5], which was in line with previous studies showing that CEV-infected fish often develop secondary bacterial infections [18]. However, in the study performed by Zhou and coworkers (2025) microbiota in CEV-infected koi was presented only as a relative abundance and in the gills only [5]. Even though CEV impacts mostly the gills, microbiota analysis would be incomplete without intestine data, since stress response induced during CEV infection might also affects the gut microbiota.

The factor that cannot be overlooked when talking about infection- and/or stress-induced microbiota dysbiosis is integrity of epithelial barrier. It is maintained by tight junction (TJ) proteins, such as claudins,and occludin, and desmosomes [19]. TJ are involved both in maintaining integrity of epithelium and regulation of its selective permeability. Changes in the expression of genes encoding TJ proteins were described both as a cause and consequence of microbiota alterations. Microbial metabolites, pathogens, and host stress responses can modulate epithelial barrier integrity, leading to increased permeability, immune activation, and other systemic effects [20]. Environmental factors e.g. salinity are well-known to affect expression and function of TJ [21]. Furthermore, cortisol was reported to increase expression of gene encoding occludin and decrease paracellular permeability in the primary culture of rainbow trout gill epithelium [22]. However, chronic exposure to stress had a different outcome, for example it decreased expression of genes encoding occludin and ZO-1 in the midgut of gibel carp [11]. Moreover, changes in the expression of TJ proteins were observed upon viral infections e.g. cyprinid herpesvirus 3 (CyHV-3) infection of common carp led to an increased expression of genes encoding claudins-2, -3c, -11, and -23 in the intestine and their higher expression was correlated with antiviral and proinflammatory response [23]. CyHV-3 was also reported to damage the skin barrier and during infection downregulation of expression of genes encoding mucin 5B and claudins 23 and 30 was observed, which could lead to secondary bacterial infections [24].

The aim of this study was to further explore the effects of stress during CEV infection on microbial community composition of koi, alongside the associated histopathological changes in the gills and two parts of the gut: the foregut and the hindgut. In addition, we studied immune and mucosal response and expression of genes encoding TJ proteins. Our study helps to understand the effect of CEV infection on the host microbiota and partly explains the mechanisms for occurrence of secondary bacterial infections related to KSD.

## Materials and methods

### Fish

Naive koi were obtained as feeding yolk sac fry from the University of South Bohemia in Ceske Budejovice, Faculty of Fisheries and Protection of Waters, located in Vodnany, Czech Republic. Fish were raised and kept under virus- and parasite-free conditions in an indoor aquaculture recirculation system at 20 °C. Fish were fed a commercial feed (Perla Plus, Skretting Norway) at a ratio of 1% body weight per day.

### Ethics

All experiments were carried out in accordance with national and international regulations for experimentation with animals under approval of the 2^nd^ Local Ethics Committee in Krakow, Poland (no. 355/2021).

### Infection experiment

Fish (12 months old koi, mean weight = 36 g) were experimentally infected with carp edema virus by cohabiting naive koi (*n* = 6) with three CEV-positive donor fish. For this study CEV from genogroup IIa was used. For the mock-infected group, koi (*n* = 6) were cohabitated with a noninfected fish and used as experimental controls. At 6 days post-infection (dpi) fish from CEV-infected and mock-infected (control) groups were euthanized by immersion in 0.5 g L^–l^MS-222 (Sigma, USA) for sample collection.

### Sample collection

Blood was collected with S-Monovette (Sarstedt, Germany) from dorsal aorta. Second left gill arch was collected into 100% ethanol (HPLC) and placed in −20 °C for microbiota analysis. Third left gill arch was collected into RNA later for viral load and gene expression analysis. Fourth left gill arch was placed in Bouin’s solution (Sigma-Aldrich, MO, USA) for 48 h until further processing for histological analysis. Whole intestine was dissected from the fish and samples were collected from the foregut (behind the intestinal bulb) and the hindgut (1 cm before the end of the intestine). In both cases, three parts of gut were collected: (i) with food content into Bouin’s solution (for 48 h until further processing) for histological analysis; (ii) without food content into RNA later for viral load and gene expression analysis; (iii) without food content into 100% ethanol (HPLC) (placed in −20 °C) for microbiota analysis. Moreover, for microbiota analysis water samples from each tank were collected and frozen immediately.

### Cortisol and glucose measurements

Cortisol and glucose concentrations were measured in the blood plasma. Cortisol level was measured with the immunoenzymatic assay Cortisol ELISA (Neogen, USA) according to the manufacturer’s protocol. Glucose level was measured with the use of glucose strips and glucometer iXell® OLED (Genexo, Poland).

### Histological analysis

The gills, foregut, and hindgut samples were collected into Bouin’s solution.

After 48 h, fixed samples were dehydrated in graded ethanol solutions starting from 70% ethanol and placed in clearing agent—Xylen (Sigma-Aldrich, MO, USA). Samples were embedded in Paraplast Plus (Leica, Germany) and cut into 6 µm sections using microtome Hyrax M55 (Zeiss, Germany). Sections were stained with hematoxylin and eosin (H and E staining). Selected sections were stained with Periodic Acid-Schiff (PAS) Alcian Blue at pH 2.5 to visualize goblet cells and mucins. Sections were visualized with Nikon Eclipse E600 microscope with  10× to 40× objective magnifications, digitalized using the NIS-Elements F software.

## Amplicon-based microbiota screening

### DNA extraction and amplicon libraries preparation

The composition and abundance of the microbiota were analyzed on the basis of V4 region of 16S rRNA gene. DNA was extracted from the gills and two distinct parts of the gut (foregut and hindgut) from control and experimental fish using custom magnetic beads-based protocol [25]. In the first step, the sample underwent mechanical homogenization using ceramic beads with the OMNI Bead Ruptor Elite homogenizer. The second step involved chemical lysis using the “Vesterinen” lysis buffer and proteinase K. After a 2-h incubation at 55 °C, the homogenate was purified using SPRI magnetic beads. During this stage, 20 000 copies of a linearized Ec5002 plasmid carrying an artificial 16S rRNA amplification target were added to each homogenate aliquot for subsequent quantification and estimation of the total bacterial absolute abundance [26]. At the DNA extraction stage, one positive and two negative controls were included. Amplicon libraries were prepared using a two-step PCR approach. In the first round of PCR (25 cycles), the V4 region of 16S rRNA gene was amplified using specific primers 515F/806R [27] with Illumina adapter stubs. PCR products were verified using agarose gel electrophoresis and purified using SPRI beads. The bead-purified PCR products were used as the template for the second indexing PCR reaction (7 cycles) during which unique combinations of index sequences were added to each sample. Two positive and two negative (blank) controls were included in PCR I, and an additional blank control was included in PCR II. Following the second agarose gel electrophoresis, all amplicon libraries were pooled on the basis of band intensity, cleaned with SPRI beads, and submitted for sequencing on NextSeq 2000 P2 600-cycle flow cell.

### Analysis of amplicon sequencing data

Amplicon data were analyzed using a custom pipeline [28] based on USEARCH/VSEARCH [25]. Reads assembled into contigs were quality-filtered, then dereplicated and denoised, aligned against the SILVA v. 138 [29] database, screened for chimeras using UCHIME [30], classified taxonomically, and clustered at 97% identity level using the nearest-neighbor algorithm and divided into operational taxonomic units (OTUs). The obtained OTU table was then decontaminated using a custom script that excluded any OTUs taxonomically assigned as mitochondria, chloroplasts, Eukaryota, Archaea, or chimeras. At this stage, potential contaminants, including reagent-derived taxa (e.g., *Brachybacterium*), were also excluded by comparing the maximum relative abundance of each unique 16S rRNA OTU in experimental samples with that in negative controls. OTUs whose maximum relative abundance in experimental libraries was less than ten times that observed in blank controls were classified as contaminants and removed from the final OTU table. The script also removes the spike-in’s reads from the final OTU Table. In addition, after the initial decontamination based on the negative controls, amplicon data were subsequently critically checked, and OTUs representing bacteria present in the tank water were removed from the dataset. The absolute abundance of bacterial 16S rRNA gene copies in each processed sample was estimated on the basis of the spike-in reference [26]. The ratio of decontaminated reads to spike-in reads was multiplied by the number of spike-in copies added (20,000) and by five, accounting for the use of only one-fifth of the insect homogenate during DNA extraction. Data on the absolute abundance of microbiota were calculated in Microsoft® Excel® and visualized using Processing 3 software v. 3.5.4. Vennplots were generated using RStudio. Inkscape 1.3.2 was used to modify generated plots and visualizations. Sequence data have been deposited in GenBank under BioProject number PRJNA1295704.

### RNA isolation and cDNA synthesis

RNA was isolated from the gills, foregut and hindgut samples as described previously [3] using TRI reagent (Sigma, USA) according to the manufacturer’s protocol; and from the foregut and hindgut as described previously [10] using GeneMATRIX Universal RNA Purification Kit (EURx, Poland). Samples were homogenized with FastPrep-24™5G (MP Biomedicals, New Zeland), then total RNA was isolated using the aforementioned commercial kits. To purify samples from any genomic DNA contamination, samples were subjected to additional DNase I (2 U) digestion (Thermo Fisher Scientific, USA). cDNA was synthesized from 1000 ng of RNA using High-Capacity cDNA Reverse Transcription Kit (Applied Biosystems, CA, USA) according to the manufacturer’s protocol.

## Real-time quantitative PCR

### Viral load analysis

DNA was isolated from the gills, foregut, and hindgut. Samples were homogenized with FastPrep-24™5G (MP Biomedicals, New Zeland) and DNA was extracted using the QIAamp DNA Mini Kit (Qiagen, Germany) according to the manufacturer’s protocol. Probe-based RT-qPCR (TaqMan) was used to determine viral load in the gills, foregut, and hindgut. P4a gene was targeted to quantify viral load [31]. Viral DNA copies were normalized per 250 ng of DNA.

### Gene expression analysis

SYBR ®Select Master Mix (Applied Biosystems, CA, USA) was used to determine gene expression. RT-qPCR was performed using Rotor-Gene Q, 5-Plex HRM (Qiagen, Germany) as described previously [10]. 40S ribosomal protein S11 and elongation factor 1 alpha were used as reference genes. Primers, used at 1 µM concentration, are presented in Additional file 1, alongside accession number. Gene expression is presented as a ratio of reference genes encoding the 40S ribosomal protein S11 (40 s) and the elongation factor 1 alpha (ef1a) to target genes using the Pfaffl method:

$${\mathrm{Ratio}} = E^{Ct} \,{\mathrm{Reference}} / E^{Ct} \,{\mathrm{Target}}$$

where *E* is the amplification efficiency, and *Ct* is the threshold cycle [32].

### Statistical analysis

Statistical analysis was done in GraphPad Prism v. 10. Normal distribution of the samples was assessed by Shapiro–Wilk test. Significant differences (*p* < 0.05) in blood parameters and gene expression studies were assessed using Student’s *t*-test in cases when the data were normally distributed and variances were homogenic or by Mann–Whitney *U* test when the data were not normally distributed.

## Results

### Viral load and stress response

**Figure 1:**
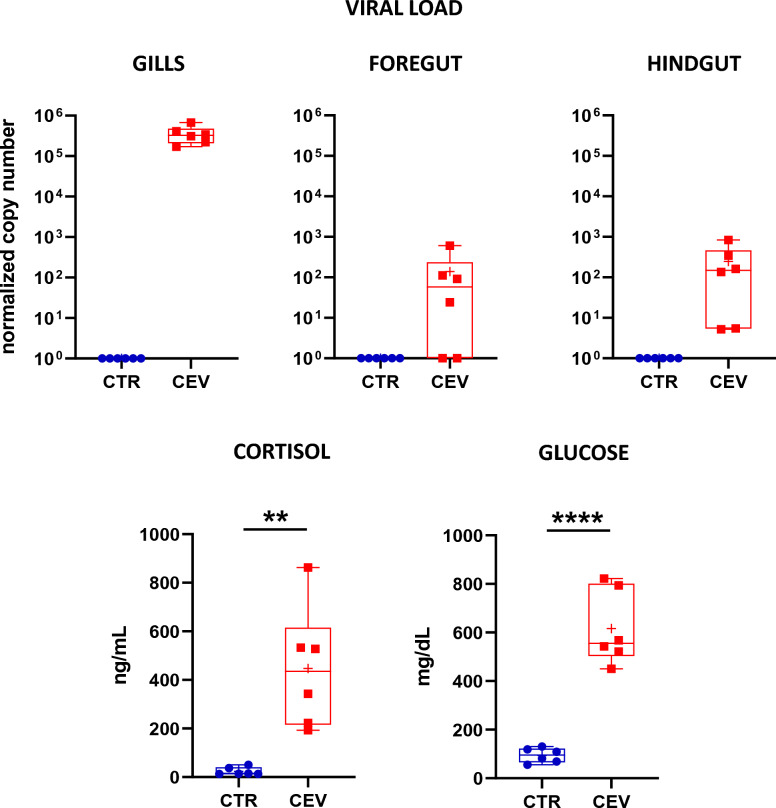
**Viral load and level of cortisol and glucose in koi 6 days post-infection with CEV.** Viral load is presented as viral DNA copies normalized per 250 ng of DNA in the gills, foregut, and hindgut. Level of cortisol [ng/mL] and glucose [mg/dL] was analyzed in the blood plasma. For the stress markers statistical analysis was performed by Student’s *t*-test or Mann–Whitney *U* test. Asterisks indicate statistically significant differences between control and infected fish (**p < 0.01; ****p < 0.0001). The data are presented as *n* = 6 individual values on box and whiskers plot with “+” as a mean.

To confirm the infection, viral load was analyzed in studied organs. Infected fish had mean 3.53 × 10^5^ viral DNA copies in the gills, 1.4 × 10^2^ DNA copies in the foregut and 1.49 × 10^3^ DNA copies in the hindgut (Figure 1). To confirm stress response, cortisol and glucose levels were measured in the blood plasma. CEV infection resulted in elevation of cortisol level from mean 23.58 ng/mL in control fish to 446.67 ng/mL in infected fish. Glucose level increased from mean 93.83 mg/dL in control fish to 616.33 mg/dL in infected fish (Figure 1). Our results confirm that the gills are the main organs for CEV replication and CEV infection induces stress response in koi.

### Histopathology

**Figure 2:**
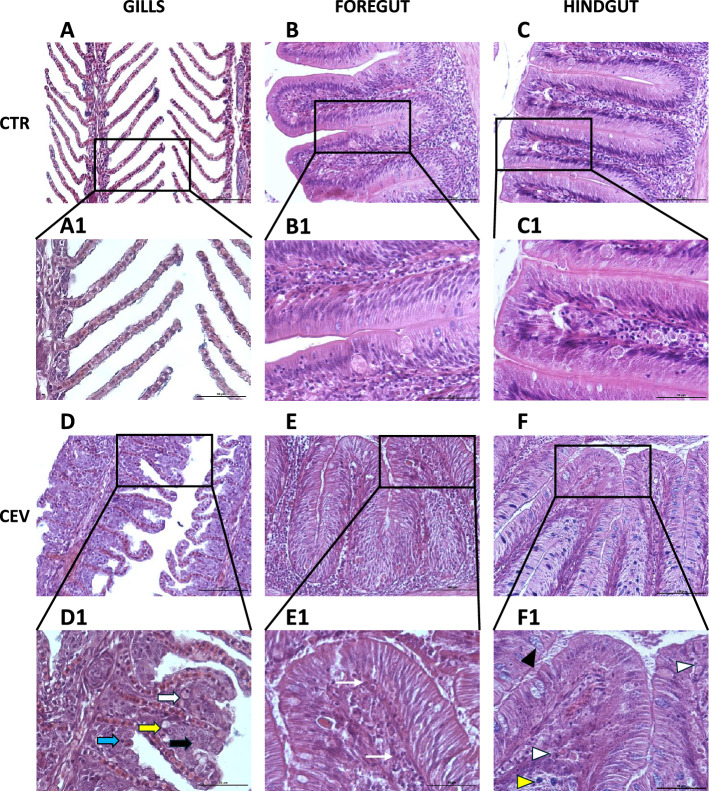
**Impact of the CEV infection on the histology of the gills, foregut, and hindgut.** Histology of the gills, foregut, and hindgut of control (**A**–**C** and **A1**–**C1**) and CEV-infected (**D**–**F** and **D1**–**F1**) koi. **D1** White arrow—epithelial cell showing pyknotic nucleus, yellow arrow—necrosis, blue arrow—apoptotic body, black arrow—epithelial hyperplasia and lamellar fusion. **E1** Narrow white arrows—lymphocytes. **F1** Black triangle—rodlet cell, white triangles—eosinophilic granular cell, yellow triangle—goblet cell. Representative pictures from *n* = 3. Bar 100 μm (**A**–**F**) and 50 μm (**A1**–**F1**).

In the gills, CEV infection resulted in hyperplasia and hypertrophy of respiratory epithelium, presence of apoptotic bodies, and focal necrosis (Figure 2D, panel D1) which were not observed in the gills of control fish (Figure 2A, panel A1).

The intestinal mucosa was composed of a monolayer of columnar epithelium with goblet cells and rodlet cells present (Figure 2B, C, B1, C1, E, F, E1, F1). Goblet cells in the intestine contained mostly acid mucins and some neutral mucins based on PAS/AB staining (data not shown). Blue staining with H and E suggested that the acid mucins were carboxylated (Figure 2F). Rodlet cells were more prominent in the hindgut. Other cells included intraepithelial lymphocytes and some eosinophilic granular cells. McKnight cells were abundant in some infected individuals, sometimes being shed into the lumen. Lamina propria contained lymphocytes and eosinophilic granular cells particularly in infected fish. Reasonably large numbers of lymphocytes, eosinophilic granular cells were present in submucosa in some infected individuals (Figure 2E1, F1).

### Gill and gut microbiota

The composition and abundance of the microbiota were determined on the basis of sequencing of the V4 region of the bacterial 16S rRNA gene. After all steps of analysis, including decontamination using negative controls and tank water, a total of 337,516 reads were obtained from 31 libraries. Reads corresponding to low-abundance OTUs, defined as those with less than 0.01% of total reads or present only in individual fish, were excluded from further analysis. The final dataset represented 95.7% of the total reads. Microbiota diversity in control and CEV-infected fish was analyzed at both the phylum and genus levels, separately for the gills, foregut, and hindgut. The comparisons focused on the five most abundant bacterial phyla and the top 20 genera selected for particular organs.

**Figure 3:**
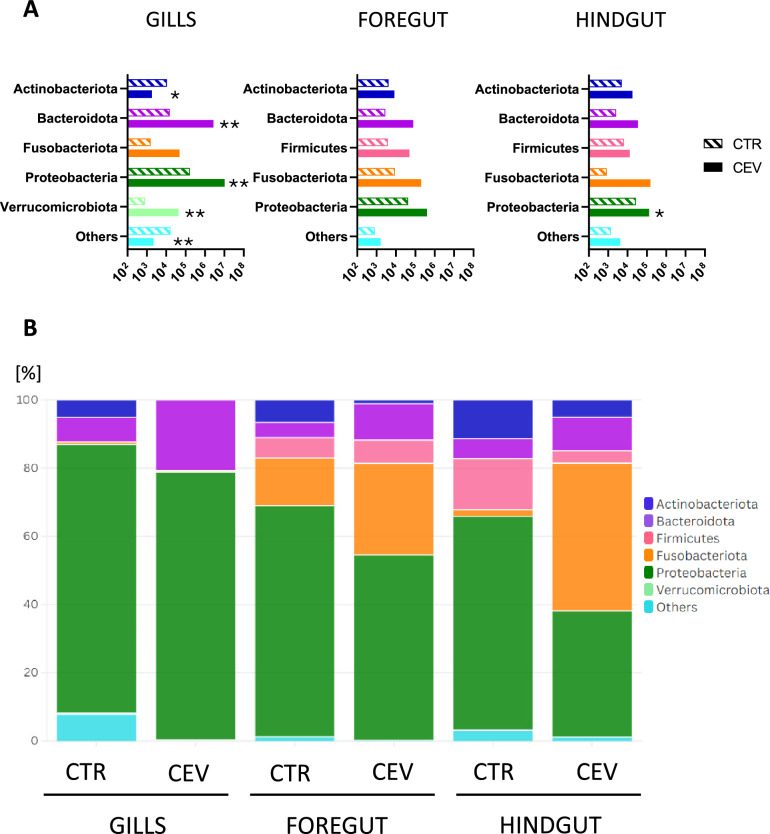
**Impact of CEV infection on the gills, foregut, and hindgut microbiota.** **A** Comparison of absolute abundance of bacterial phyla in the gills, foregut, and hindgut of control (CTR, *n* = 4) and CEV-infected (CEV, *n* = 6) koi. Statistical analysis was performed by Student’s *t*-test or Mann–Whitney *U* test. Asterisks indicate statistically significant differences between control and infected fish (*p < 0.05; **p < 0.01). **B** Relative abundance of bacterial phyla in the gills, foregut, and hindgut of control and CEV-infected koi.

The microbiota of the gills was dominated by the bacteria belonging to the phyla: Actinobacteriota, Bacteroidota, Fusobacteriota, Proteobacteriota, and Verrucomicrobiota. In both the foregut and hindgut, the five most abundant phyla were: Actinobacteriota, Bacteroidota, Firmicutes, Fusobacteriota, and Proteobacteriota (Figure 3 and Additional file 2). Amplicon analyses revealed that CEV infection affected the microbiota composition of the gills and hindgut. In the gills of CEV-infected fish absolute abundance of Bacteroidota, Proteobacteria, and Verrucomicrobiota was increased, while abundance of Actinobacteriota and other phyla was decreased. In the hindgut, the increased abundance of Proteobacteria in CEV-infected fish was observed compared with control fish (Figure 3). In contrast, in the foregut, no significant changes in the microbiota composition were detected at the phylum level between control and CEV-infected koi (Figure 3).

**Figure 4:**
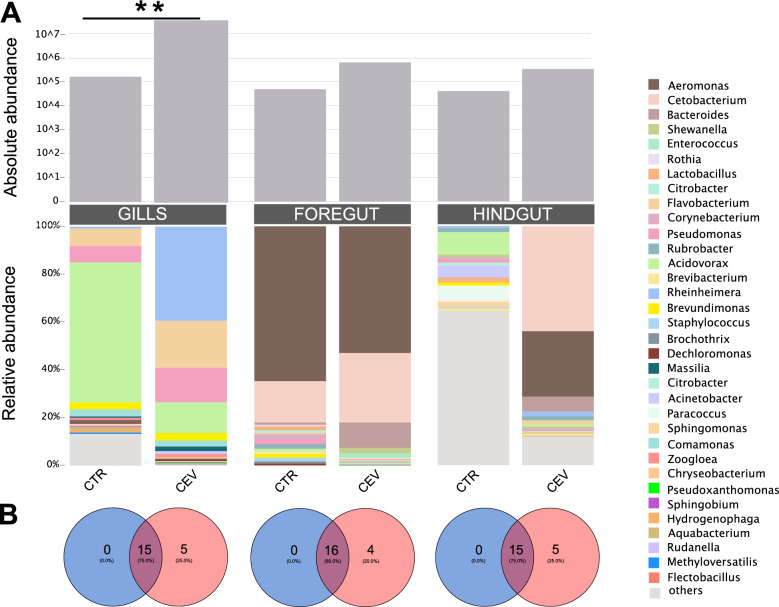
**Microbiota composition and bacteria abundance in the gills, foregut, and hindgut of control and CEV-infected koi.** **A** OTUs outside the top 20 most abundant, but with an absolute abundance greater than 0.01% of reads in a given library, were classified as “others”. Statistical analysis was performed by Student’s *t*-test or Mann–Whitney *U* test. Asterisks indicate statistically significant differences in absolute abundance of bacteria between control and CEV-infected koi (**p < 0.01). **B** Venn plots showing the overlap of the top 20 bacterial genera between control and infected fish in the gills, foregut, and hindgut, respectively. The data are presented as mean from *n* = 4 in case of CTR and *n* = 6 in case of CEV.

At the genus level, the total number of bacteria genera revealed in control and CEV-infected koi was 41 in the gills, 84 in the foregut, and 157 in the hindgut. An overall increase in the absolute abundance of microbiota was observed in CEV-infected fish compared with the control ones (Figure 4A). Venn plots showed that in all analyzed organs, CEV-infected fish harbored four (in the foregut) or five (in the gills and hindgut) bacterial genera that were not detected in control fish, indicating that the infection may promote the colonization of specific bacterial taxa absent in healthy fish (Figure 4B).

**Figure 5:**
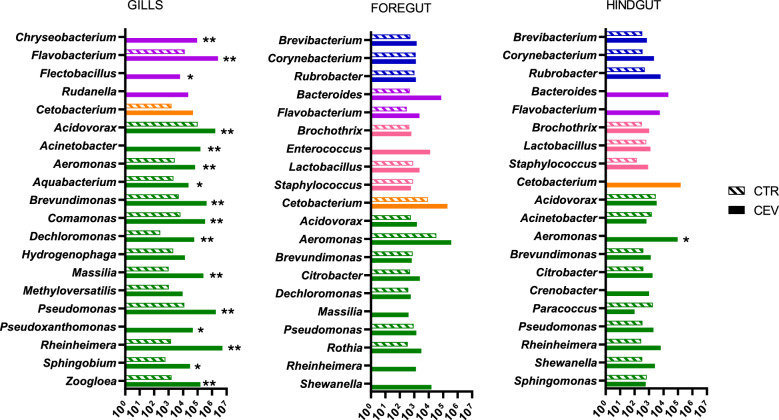
**Impact of the CEV infection on the gills, foregut, and hindgut microbiota.** Comparison of absolute abundance of bacterial genera in the gills, foregut, and hindgut of control and CEV-infected koi. Colors represents different phyla: Actinobacteriota (blue), Bacteroidota (purple), Firmicutes (pink), Fusobacteriota (orange), and Proteobacteriota (green). Statistical analysis was performed by Student’s *t*-test or Mann–Whitney *U* test. Asterisks indicate statistically significant differences between control and infected fish (*p < 0.05; **p < 0.01) (*n* = 4 in case of CTR and *n* = 6 in case of CEV).

The gills appeared to be the organ most affected by the infection. Among the top 20 most abundant bacterial genera, 16 showed significant increase in abundance in the gills of CEV-infected fish, including the genera *Chryseobacterium, Flavobacterium*, and *Flectobacillus* (all belonging to phylum Bacteroidota) and *Cetobacterium* (phylum Fusobacteriota) (Figure 5). In the gut, most of the top 20 most abundant bacterial genera belonged to the phylum Proteobacteria. In the hindgut of CEV-infected fish, the abundance of only *Aeromonas* was significantly increased (Figure 5)*.* Several genera including: *Bacteroides, Flavobacterium, Cetobacterium, Aeromonas,* and *Crenobacter* were detected in the hindgut of CEV-infected fish and were not present in the hindgut of control fish (Figure 5). In the foregut, we did not observe statistically significant alterations in the gut microbiota (Figure 5).

**Figure 6:**
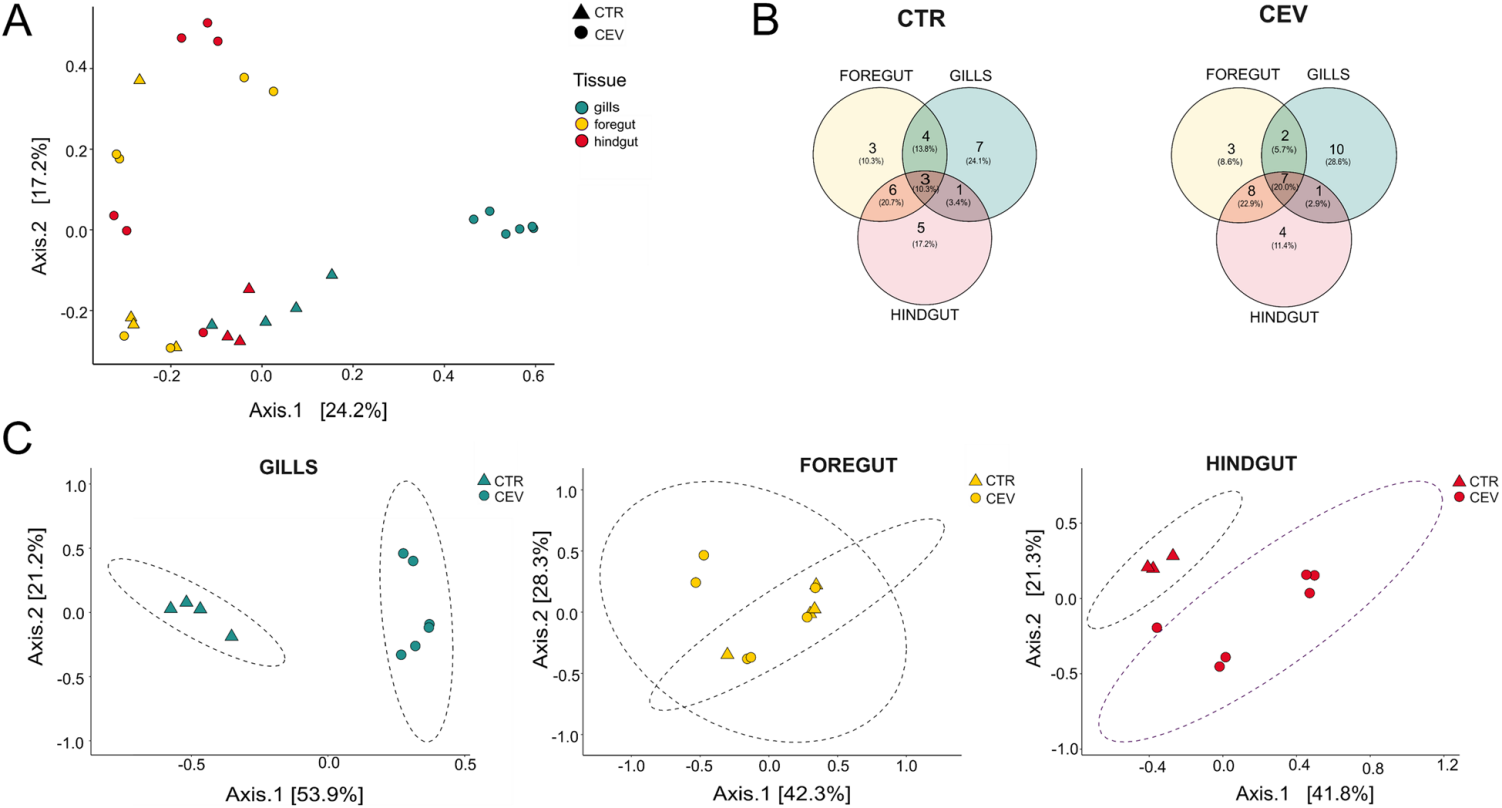
**Impact of the CEV infection on the gills, foregut and hindgut microbiota.** **A** Principal coordinates analysis (PCoA) of Bray–Curtis distances showing differences in microbiota composition between the gills, foregut, and hindgut in control and CEV-infected koi. **B** Venn plots showing the overlap of the top 20 bacterial genera between the gills, foregut, and hindgut, in control and infected koi, respectively. **C** PCoA based on Bray–Curtis distances showing differences in microbiota composition between control and infected koi in the gills, foregut, and hindgut, respectively. The data are presented as individual values from *n* = 4 in case of CTR and *n* = 6 in case of CEV.

Beta diversity analysis revealed a significant effect of CEV infection on microbial community composition across all organs. Using Bray–Curtis dissimilarity and a permutational multivariate analysis of variance (PERMANOVA) test, we found that treatment explained 8.3% of the variation in community structure (*R*^2^ = 0.083, *p* = 0.01). Although the proportion of explained variance was modest, the result suggests that CEV infection had a significant influence on the overall microbial community composition (Figure 6A). Overall, the distribution of the top 20 bacterial genera across the analyzed organs in the control and CEV-infected fish revealed that, the gills of CTR fish harbored the highest number of unique bacterial genera (*n* = 7, 24.1%), followed by the hindgut (*n* = 5, 17.2%) and the foregut (*n* = 3, 10.3%). Six bacterial genera (20.7%) were shared between the foregut and hindgut, while only three (10.3%) were common to all three tissues (Figure 6B). In contrast, CEV infection led to a marked increase in unique bacterial genera in the gills (*n* = 10, 28.6%) and an increase in those shared across all organs (*n* = 7, 20%). Notably, the overlap between the foregut and hindgut microbiota increased slightly (*n* = 8 bacterial genera, 22.9%) under CEV exposure, while shared bacterial genera between the gills and foregut were reduced (Figure 6B). In particular, beta diversity analysis showed organ-specific differences in microbial community structure in response to CEV infection (Figure 6C). PERMANOVA based on Bray–Curtis dissimilarity indicated that CEV infection had a significant effect on the microbial communities in both the gill (*R*^2^ = 0.528, *p* = 0.004) and hindgut (*R*^2^ = 0.315, *p* = 0.042), explaining 52.8% and 41.8% of the variance, respectively. In contrast, no significant differences were observed in the foregut microbiota (*R*^2^ = 0.094, *p* = 0.545), suggesting that the microbial composition in this region is less responsive to the infection.

### Gene expression

**Figure 7:**
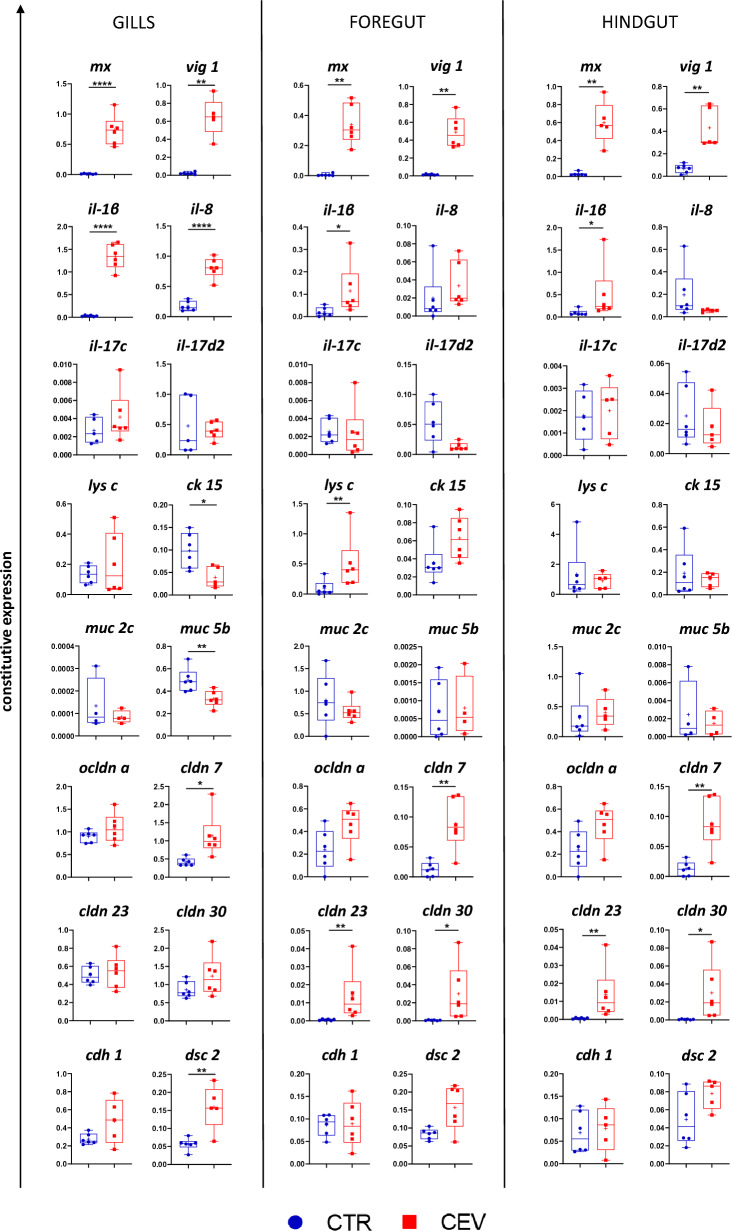
**Impact of the CEV infection on constitutive gene expression in the gills, foregut, and hindgut of koi.** Statistical analysis was performed by Student’s *t*-test or Mann–Whitney *U* test. Asterisks indicate statistically significant differences between control and infected fish (*p < 0.05; **p < 0.01; ****p < 0.0001). The data are presented as *n* = 6 individual values on box and whiskers plot with “+” as a mean. Blue dots mark control fish and red squares mark infected fish.

CEV infection resulted in significant upregulation of expression of antiviral *mxa, vig 1* and proinflammatory *il-1b* in the gills, foregut, and hindgut. Expression of *Il-8* was upregulated only in the gills of CEV-infected fish. Expression of *il-17c* and *il-17d2* did not change during infection. Expression of *lys c,* encoding anti-bacterial lysozyme C, was upregulated in CEV-infected koi but only in the foregut. Gene encoding cytokeratin 15, which is a key components of intermediate filaments in epithelial cells and is involved in the maintaining of epithelial cell integrity, was downregulated in the gills of the infected fish. Mucosa-related genes were also studied. *Muc 5b* was downregulated in the gills of infected fish. Expression of *muc 5b* in the foregut and hindgut and expression of *muc 2c* in all studied organs were not affected by infection, while expression of *cldn 7* was upregulated in all studied organs upon CEV infection. Expression of *cldn 23* and *cldn 30* was upregulated upon infection in the foregut and hindgut. Expression of *ocldn a* and *cdh 1* was not affected by the viral infection. In CEV-infected fish, desmocollin 2 encoding gene was upregulated only the gills (Figure 7).

## Discussion

In fish, as in other vertebrates, viral infections can be accompanied by bacterial coinfections, which may lead to additional pathogenesis [18, 33–36]. Bacterial coinfections were reported during infection with two different fish poxviruses: CEV [18] and salmon gill poxvirus (SGPV) [37, 38]. In case of CEV, coinfection with *Flavobacteria* was observed, which could be responsible for exacerbation of clinical signs exhibited by fish with KSD [18, 39]. In the case of SGPV-infected fish, microbial pathogens such as *Candidatus Branchiomonas cysticola* and *Ca. Piscichlamydia salmonis* were observed; however, it remains a matter of debate which of the pathogens is the primary causative agent of the complex gill disease [38]. These observations led us to closer evaluation of changes in bacterial communities in the gills, foregut, and hindgut of koi during CEV infection.

Recent studies of gill microbiota in two groups of CEV-infected koi (early-infected and late-infected) and control fish, revealed the top seven dominant phyla in all studies groups: Actinobacteriota, Bacteroidota, Firmicutes, Fusobacteriota, Proteobacteria, Verrucomicrobiota, Chlamydiae, and unidentified bacteria [5]. During the infection, significant modifications were observed with decreased abundance of Proteobacteria and Fusobacteriota in early-infection group when compared with control fish and increased abundance of Bacteroidota in early-infection group when compared with the late-infection group [5]. However, it needs to be emphasized, that the abovementioned study by Zhou and coworkers [5] presented gills microbiota component only as a relative abundance. We have expanded our study by analysis of microbiota of the gills, foregut, and hindgut and presented the results as absolute abundance. We observed the same most dominant phyla in the gills, foregut, and hindgut of control and CEV-infected fish as those described for the gills by Zhou and coworkers [5]. In CEV-infected fish, dysbiosis occurred foremost in the gills, which are the primary target for the virus. Meanwhile, in the foregut and hindgut, where the viral load was lower, we did not detect many changes in the absolute abundance of bacteria. This is in accordance with a previous study regarding rainbow trout infected with IHNV, where microbial dysbiosis in the digestive track was related to viral load and intensity of the immune response [16].

When studying the changes of microbial community composition in the gills of CEV-infected koi on the genus level, Zhou and coworkers [5] revealed differences only between fish from early-infection and late-infection groups and showed higher abundance of *Hydrogenophaga* and *Flavobaterium* in fish from the early-infection group. Our study revealed increased absolute abundance of 16 bacterial genera in the gills, one in the hindgut, and no changes in the foregut. From the phylum Bacteroidota higher absolute abundance in the gills of CEV-infected fish was noted for *Chryseobacterium, Flavobacterium*, and *Flectobacillus*. Both *Chryseobacterium* and *Flavobacterium* are fish pathogens involved in flavobacterial infections [40]. However, *Chryseobacterium* could be fish commensal as well [40]. *Flectobacillus* was mostly isolated from freshwater [41], although *Flecotbacillus roseus* was reported to induce flectobacillosis in captive held carp *Labeo rohita* [42]. In the case of Proteobacteria, CEV infection induced an increased absolute abundance of many opportunistic or pathogenic bacteria, including *Acinetobacter* [43], *Aeromonas* [44], and *Pseudomonas* [45, 46]. In addition, increased absolute abundance was observed for bacteria from genera associated with freshwater and/or biofilms and sediments e.g.: *Acidovorax* [47, 48], *Aquabacterium* [49], *Brevundimonas* [47, 50], *Comamonas* [51, 52], *Dechloromonas* [53], *Massilia* [54], *Pseudoxanthomonas* [55], *Rheinheimera* [56], *Sphingobium* [57, 58], and *Zoogloea* [59]. In the hindgut of CEV-infected fish, we observed the occurrence of five bacteria genera: *Bacterioides, Flavobaterium, Cetobacterium*, *Aeromonas*, and *Crenobacter,* which were not detected in the hindgut of control fish. Previous studies showed that in common carp infected with SVCV, relative abundance of *Cetobacterium* and *Aeromonas* was increased alongside *Shewanella* in the gut mucosa when compared with control fish. Such microbiota changes were associated with pathology and could lead to altered nutrition absorption [14]. In turn, cyprinid herpesvirus 2 (CyHV-2) infection of Prussia carp (*Carassius gibelio*) altered gut microbiota, with the highest increase in relative abundance in Proteobacteria, in particular *Aeromonas* [60] which is in line with our results, while the relative abundance of *Cetobacterium* decreased during infection with CyHV-2 [60]. In our study *Cetobacterium* was detected in the foregut of the control and CEV-infected fish while in the hindgut it was present only in CEV-infected fish, suggesting translocation of these bacteria to the hindgut during infection. Relative abundance of *Cetobacterium* increased across the intestine of grass carp (*Ctenopharyngodon idellus*) infected with GCRV [8]. Although, *Cetobacterium* are intestinal anaerobic bacteria involved in vitamin B12 production [61], their growth during infection could be related to hypoxic conditions present during infection [8]. This could explain its increase during CEV infection, given hypoxia is one of the clinical signs of KSD [3].

As mentioned above, during viral infections, coinfections with pathogenic *Flavobacteria* are often observed and can exacerbate clinical signs of the disease or even increase mortality. Coinfection with *Flavobacteria* was found during infection with CEV [18, 62] and other viruses such as CyHV-3 [24]; CyHV-2 [63]; largemouth bass virus (LMBV) [64]; IHNV [7, 65]. Interestingly, the increase of *Flavobacteria* abundance was previously connected with a decrease in the abundance of *Pseudomonas* [16, 66]. *Pseudomonas* was reported to control growth of opportunistic bacteria e.g. *Flavobacteria* [66]. However, in the current study, we observed an increased absolute abundance of both *Flavobacteria* and *Pseudomonas* in the gills of infected koi. Recently Klak and coworkers (2025) demonstrated an increased abundance of *Flavobacterium* in the gut of antibiotic-treated carp [10].

In this and our previous studies [4], we demonstrated that CEV infection leads to elevated cortisol levels in the blood plasma. In this context, we cannot exclude that an increased cortisol level can be involved in microbiota changes in CEV-infected fish. This phenomenon of stress-induced gut microbiota dysbiosis was previously described in Atlantic salmon [9]. This study revealed increased abundance of *Acinetobacter* and *Aeromonas,* both after acute and chronic stress [9]. Stress can have a different outcome depending on whether it is acute or chronic. In common carp gut, acute prolonged restraint stress decreased *Cetobacterium* abundance and increased *Vibrio* abundance [10], while chronic stress in gibel carp resulted in increased abundance of *Cetobacterium* [11]. Moreover, stress induced after antibiotic treatment resulted in increased abundance of *Mycoplasma, Polynucleobacter*, and *Pseudomonas* in common carp [10]. Cortisol can impact on coinfections in various ways, whether owing to a direct impact on the bacteria growth or owing to immunosuppression of the host. Interestingly, cortisol was previously reported to facilitate *Flavobacteria* growth [67, 68], giving a probable explanation for the flavobacterial coinfections observed during KSD.

Furthermore, disruption in microbiota may be owing to the immunomodulatory nature of CEV. In a previous study, we found that CEV downregulated the expression of several adaptive immunity genes, as well as neutrophil marker *mpo* in the gills [4]. Modulation and suppression of the immune response was also shown in mammalian poxviral infections [69] and fish poxviruses [70]. This combined with the stress during infection can potentially make fish more susceptible to bacterial infections, especially since a variety of commensal bacteria harboring mucosa can be facultative pathogens [71].

We analyzed the expression of selected genes involved in the immune response and genes encoding tight-junction proteins in the gills, foregut, and hindgut. Expression of antiviral genes and gene encoding proinflammatory Il-1β was upregulated in all three organs, while the expression of *il-8* was upregulated in the gills only. The extent of the immune response was related to the viral load, which was higher in the gills than in the gut. In our study, *il-1β* expression was upregulated in all studied organs, with the highest constitutive expression observed in the gills, indicating strong inflammation in this organ. The strong inflammation in the gills can be linked to the changes of microbial community composition in this organ. To further investigate interactions between immune response and microbiota dysbiosis, we analyzed the expression of *il-17c* and *il-17d2*, as Th17 cells and their associated cytokines play a key role in mediating host response to infection [72]*.* However, the expression of these genes was not changed during infection with CEV. Previous study demonstrated that treatment with antibiotics alongside restraint stress upregulated expression of these genes in common carp, possibly influencing dysbiosis owing to inflammation in the gut [10]. Similarly, in grass carp, 5 day long antibiotic treatment upregulated expression of *il-17N* [73]. This indicates that dysbiosis induced by antibiotics could be exacerbated by disruption of the balance between the proinflammatory and regulatory T cell responses.

Microbiota dysbiosis is very often connected with the disruption of mucosal barriers. CEV infection affected gills, with serious histopathological changes visible. Gene expression provided more insight into the damage of epithelial cells, as cytokeratin 15 gene expression was significantly downregulated in the gills of CEV-infected fish. Meanwhile, during infection with another fish poxvirus—SGPV, expression of desmophilin 2 and claudin 4 were upregulated [70]*. *In vitro studies using different common carp derived cell lines revealed that infection with CyHV-3 resulted in a reduced expression of epithelial markers including *ck15* and *cdh* [74]*.* Infection with common carp paramyxovirus (CCPV) differently affected epithelial markers depending on the cell line; however, infection of cells with CEV did not result in replication of the virus [74]. In turn, CyHV-3 infection in vivo resulted in upregulation of expression of selected TJ protein-encoding genes in the gut [23] and downregulation of their expression in the skin [24]. During CEV infection, we observed significant upregulation of the gene expression of claudins (*cldn 7*, *cldn 7, cldn 23*). Studies on common carp infected with SVCV revealed that during infection, the gene expression of 31 cell adhesion molecules were upregulated in the gills, while only 11 were downregulated. Similarly, in the gut, 42 genes coding cell adhesion molecules were upregulated and 12 were downregulated [75]. These results suggest that viral infections cause remodeling of the epithelium [23], especially in the organs which are not the target organ for the virus. Increased expression of genes involved in maintaining epithelial barrier integrity protects or counteracts damage, which could result in inflammation and infiltration of bacteria into underlying connective tissue [76]. As fish gills and gut epithelia are covered with mucus, we analyzed the expression of two selected mucins in control and infected fish: *muc 2c, muc 5b*. Mucin 2c is highly expressed in the gut, while mucin 5b is mainly expressed in the skin and gills [77]. Previous studies on CEV-infection did not reveal significant changes in expression of *muc 2c* in the gut and *muc 5b* in the gills (5 days post-infection), while the authors of these studies observed downregulation of expression of *muc 5b* in the skin of infected koi [78]. Our research showed downregulation of *muc 5b* in the gills of CEV-infected fish, even though overproduction of mucus was observed during KSD [2]. This shows impairment of mucosal barrier in spite of the increased amount of mucus. Similarly, during CyHV-3 infection *muc 5b* was downregulated in the skin of infected carp [24], while there were no changes in the *muc 2* expression in the gut [23]. Another study by Adamek, et al. analyzed expression of different mucins in common carp infected with CyHV-3, SVCV, or CEV [79]. Interestingly, in the gills, downregulation of *muc 2-like* was observed at the onset of clinical signs during infection with all three viruses while expression of *muc 13* was downregulated but only in the gut of CyHV-3-infected fish [79]. Histopathological analysis of common carp infected with SVCV showed an increased number of mucous cells in both the gills and gut [75]. Moreover, higher expression of *muc 2* in the gills and an increased number of goblet cells in the gills filaments and lamellae was reported during infection of rainbow trout with IHNV [6]. Mucus plays a vital role in preventing infections, enabling entrapment of pathogens [80]. Thus, altered mucin expression observed during viral infections can facilitate secondary bacterial infections.

In conclusion, complex interactions occur in the mucosal tissues of koi during KSD. CEV infection induces stress and activates immune responses, ultimately disrupting the microbiota and favoring bacterial growth. This paves the way for secondary bacterial infection. Most of these changes are observed in the gills of infected fish, as this is the primary target of the virus. However, CEV still elicited immune activation and increased expression of genes encoding TJ proteins even at a lower viral load in both the foregut and hindgut. These results demonstrate that CEV disrupts mucosal and microbial homeostasis in an organ-dependent manner. Our study sheds light on the impact of CEV infection on the host microbiota and partly elucidates the mechanisms underlying secondary bacterial infections associated with KSD.

## Supplementary Information

**Additional file** **1** **Primers used for RT-qPCR**.

**Additional file** **2** **Impact of infection with CEV on the gills, foregut and hindgut microbiota of koi.** Relative abundance of bacterial phyla in the gills, foregut and hindgut of control (*n *= 3-4) and CEV-infected koi (*n *= 6). Each bar represents individual fish.

## Data Availability

All data generated or analyzed during this study are included in this published article. Sequence data have been deposited in GenBank under BioProject number PRJNA1295704.
