# Supplementary material for: Changes in the gill and gut microbiota of koi infected with carp edema virus
Source: Vet Res. 2026 Jan 8;57:20. doi: 10.1186/s13567-025-01700-y (PMC12849644; doi:10.1186/s13567-025-01700-y)
Supplement: Supplementary file 1 — Additional file 1 Primers used for RT-qPCR. [file 13567_2025_1700_MOESM1_ESM.docx]

**Additional file 1.** Primers used for RT-qPCR

| **Gene** | **Forward primer sequence** | **Reverse primer sequence** | **Accession number** |
| --- | --- | --- | --- |
| *40S* | CCGTGGGTGACATCGTTACA | TCAGGACATTGAACCTCACTGTCT | AB012087 |
| *ef1a* | ACAACCCCAAGGCTCTCAA | CCGCCAACTTTCTTCTCAAC | AF485331 |
| *CEV p4a* | AGTTTTGTAKATTGTAGCATTTCC | GATTCCTCAAGGAGTTDCAGTAAA | OP494128 |
| *mx* | ATGACCCAGCAGAAGTGGAG | CAGGAACATTGG CAGAGATG | XM_042716965 |
| *vig* | CGCACCAGAGAGCAGAAAG | CTCAATAGGCAGCACGAAC | JX131617 |
| *il-1 β* | AAGGAGGCCAGTGGCTCTGT | CCTGAAGAAGAGGAGGCTGTCA | KC008576 |
| *il-8* | TCACTTCACTGGTGTTGCTC | GGAATTGCTGGCTCTGAATG | AB470924 |
| *il-17c* | GTGCCTGTGAAGCAAACTCG | TGCACAGGTACAGGCTACAG | XM_019125070 |
| *il-17d2* | GGAGCAGATGTTCGGGAGAC | CGAGACGCTGATTGACAGGT | LC151614 |
| *lys C* | GGGACTTGATGGCTTTGAGG | GCTGAACGCACTCTGTGG | AB027305 |
| *ck 15* | CCTCCAAGTGTCCAAGACTG | GCAAGAGTTCCCTCAAGA CC | EC393545 |
| *muc 2c* | TGACTGCCAAAGCCTCATTC | CCATTGACTACGACCTGTTTCTC | XM_042752573 |
| *muc 5b* | CAGCCCTCTTCCTCTTTCATC | CCACTCATCTTTCCTTTCTCTTC | JF343438 |
| *ocldn a* | GGCTATGGAATGGGTGGAG | CGAGCAGAATGATGAAGGTG | EC393229 |
| *cldn 7* | CTTCTATAACCCCTTCACACCAG | ACATGCCTCCACCCATTATG | JQ767155 |
| *cldn 23* | CAGAACCTCCACAACGTCTAG | CCGAGCAATGTCACAATCAAC | JQ767159 |
| *cldn 30* | CCCTCAGTTCAGACCTTCAG | TGACACCGACCTTGGATTTG | JQ767160 |
| *cdh 1* | TCAGGACAGCTCCATTCAAG | AACAGCAGAGCCAAGATTCC | EC393368 |
| *dsc 2* | TGGCTTACTTG GAGACGAG | GCCACAAGGCTACAACATCC | EC393171 |
