# Supplementary material for: Changes in the gill and gut microbiota of koi infected with carp edema virus
Source: Vet Res. 2026 Jan 8;57:20. doi: 10.1186/s13567-025-01700-y (PMC12849644; doi:10.1186/s13567-025-01700-y)
Supplement: Supplementary file 2 — Additional file 2 Impact of infection with CEV on the gills, foregut and hindgut microbiota of koi. Relative abundance of bacterial phyla in the gills, foregut and hindgut of control (n = 3-4) and CEV-infected koi (n = 6). Each bar represents individual fish. [file 13567_2025_1700_MOESM2_ESM.docx]

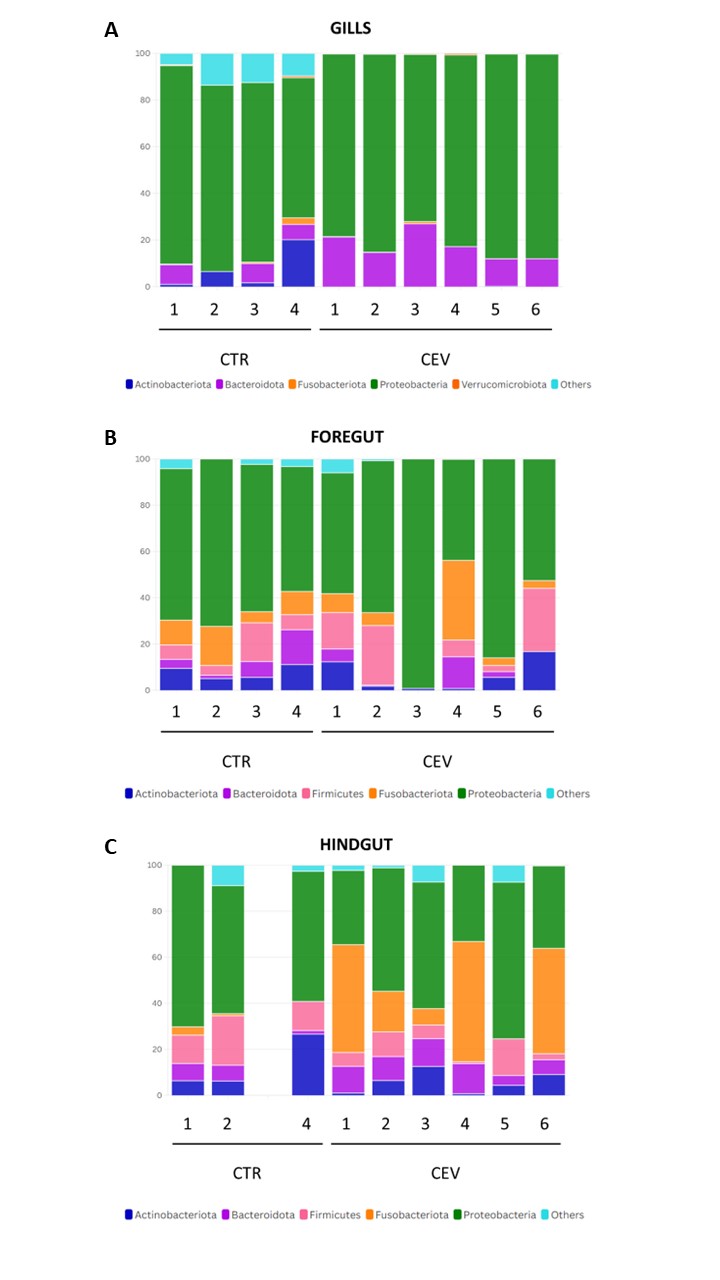


**Additional file 2. Impact of infection with CEV on the gills, foregut and hindgut microbiota of koi.** Relative abundance of bacterial phyla in the gills, foregut and hindgut of control (n=3-4) and CEV-infected koi (n=6). Each bar represents individual fish.
